# The Retinoid Tamibarotene Aggravates Skin Inflammation in a Model of Bullous Pemphigoid-like Epidermolysis Bullosa Acquisita

**DOI:** 10.3390/cells14211661

**Published:** 2025-10-23

**Authors:** Markus Thieme, Paul Schilf, Sripriya Murthy, Sina Gonther, Christoph M. Hammers, Guido Heine, Christian D. Sadik

**Affiliations:** 1 Department of Dermatology, Allergy, and Venereology, University of Lübeck, Ratzeburger Allee 160, 23538 Lübeck, Germany; markus.thieme@med.uni-rostock.de (M.T.); paul.schilf@uksh.de (P.S.);; 2 Department of Dermatology and Venereology, University of Rostock, Strempelstraße 13, 18057 Rostock, Germany; 3 Department of Dermatology, University of Regensburg, Franz-Josef-Strauss-Allee 11, 93053 Regensburg, Germany; 4 Department of Dermatology and Allergy, Christian Albrechts University of Kiel, Chrisitan-Albrechts-Platz 4, 24118 Kiel, Germany; gheine@dermatology.uni-kiel.de

**Keywords:** retinoids, tamibarotene, epidermolysis bullosa acquisita, pemphigoid diseases, autoimmune diseases, regulatory T cells, neutrophils

## Abstract

Tamibarotene (AM80) is an agonist of retinoic acid receptor alpha. It is licensed in Japan for the treatment of acute promyelocytic leukemia. Results from preclinical models suggest that tamibarotene might also be effective in the treatment of diverse autoimmune diseases. The effect of tamibarotene on autoimmune diseases of the skin, however, has not been explored. We therefore examined the effect of tamibarotene on disease in the antibody-transfer mouse model of bullous pemphigoid (BP)-like epidermolysis bullosa acquisita (EBA), a prototypical example for pemphigoid diseases. Pemphigoid diseases are a group of autoimmune blistering skin diseases driven by autoantibodies and the recruitment and activity of granulocytes in the dermis. In sharp contrast to its effect in models of other autoimmune diseases, tamibarotene aggravated EBA pronouncedly. At the peak of disease, skin inflammation in tamibarotene-treated mice involved, on average, 1.6-fold more of the total body surface compared to vehicle-treated mice. Tamibarotene markedly reduced the recruitment of regulatory T cells (T_regs_) into the dermis. This blunted the counterregulatory mechanisms that normally curb skin inflammation in this model. The effect aligns with previous reports describing tamibarotene-mediated downregulation of skin-homing receptors on T_regs_. In addition, tamibarotene prolonged the responsiveness of aging neutrophils to immune complexes in vitro, providing another mechanism that may exacerbate EBA. Collectively, our results suggest that tamibarotene may elicit detrimental effects in patients with EBA by abolishing the recruitment of T_regs_ into skin. This warrants great caution when using tamibarotene in patients with EBA and possibly other pemphigoid diseases.

## 1. Introduction

Retinoids bear complex immunomodulatory activities that are understood only partially. Their signaling is through retinoic acid receptors (RARs), a family of nuclear receptors. Tamibarotene is a retinoic acid receptor alpha (RARα) agonist. It additionally bears lower affinity towards RARβ and none to RARγ [1]. Tamibarotene is licensed in Japan for the treatment of acute promyelocytic leukemia [2]. Intriguingly, in several preclinical models of autoimmune diseases, tamibarotene suppressed tissue inflammation. This includes models of rheumatoid arthritis, ANCA-associated vasculitis, autoimmune myositis, skin inflammation in systemic sclerosis and graft-versus-host disease, autoimmune uveorenitis, autoimmune pancreatitis, renal fibrosis in chronic kidney disease, and autoimmune encephalitis [3,4,5,6,7,8,9,10,11]. Tamibarotene is therefore considered a potential new drug for the treatment of a broad spectrum of autoimmune diseases. However, the impact of tamibarotene on autoimmune diseases of the skin, such as pemphigoid diseases, is unknown.

Pemphigoid diseases are a diverse group of autoimmune blistering skin diseases [12]. They are caused by autoimmunity against different components of the dermal–epidermal adhesion complex and feature an autoantibody-induced, granulocyte-driven immune response. Herein, granulocytes are recruited into the dermis and activated there via Fcγ receptors by the immune complexes formed at the dermal–epidermal junction [12,13]. By the consequent release of leukotriene B_4_ (LTB_4_), reactive oxygen species (ROS), and proteases from neutrophils, the recruitment of granulocytes into the dermis is further amplified and the proteins of the dermal–epidermal adhesion complex are degraded [14,15,16,17,18,19]. The latter leads to the formation of subepidermal clefts, which clinically manifests as an eruption of blisters on inflamed skin. These inflammatory processes are curbed by regulatory T cells (T_regs_) recruited into the dermis [20,21].

Pemphigoid diseases are difficult-to-treat diseases associated with a high rate of mortality [22]. Novel, innovative therapeutics to treat pemphigoid diseases are consequently urgently required. The antibody-transfer mouse model of bullous pemphigoid (BP)-like epidermolysis bullosa acquisita (EBA), a pemphigoid disease caused by autoimmunity against type VII collagen, mimics the effector phase of these diseases [13,23,24]. Disease in this model is responsive to already established treatments of pemphigoid diseases [25,26]. The model was also instrumental to initiate clinical trials testing new therapeutics for these diseases, underscoring its clinical relevance for drug development for pemphigoid diseases [15,16]. Considering the therapeutic potential of tamibarotene in other autoimmune diseases, we examined the therapeutic potential of tamibarotene in this model. Anticipating a therapeutic effect of tamibarotene, we surprisingly found tamibarotene to aggravate skin inflammation pronouncedly.

## 2. Materials and Methods

### 2.1. Mice

*C57BL/6JRj* wild-type mice were purchased from Janvier Labs (Le Genest-Saint-Isle, France) and given at least two weeks to accommodate in the animal facility prior to the start of experiments. All animals were housed in a 12 h light–dark cycle in the animal facility of the University of Lübeck. All experiments were performed in 8- to 12-week-old age- and sex-matched mice by certified personnel. All experimental groups comprised an equal number of male and female mice. All experiments had been permitted in advance by the Schleswig-Holstein state government (reference number 118-9/17).

### 2.2. Antibody-Transfer Model of Bullous Pemphigoid (BP)-like Epidermolysis Bullosa Acquisita (EBA) and Treatment with Tamibarotene

**Figure 1:**
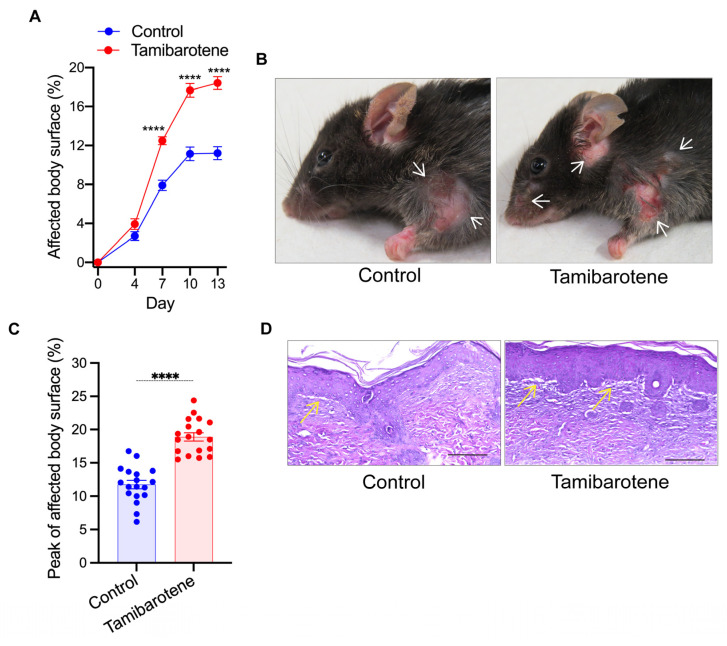
**Tamibarotene aggravates EBA.** (**A**) Percentage of the total body surface area affected by inflammatory skin lesions in vehicle control- and 3 mg/kg body weight tamibarotene-treated mice over time. (**B**) Representative pictures of vehicle- and tamibarotene-treated mice showing the clinical presentation on day 13. Arrows indicate inflammatory skin lesions. Results are presented as mean ± SEM. (**C**) Percentage of the total body surface area reached at peak of disease during the experiment in each individual mouse. Results are presented as mean ± SEM. Each dot represents a mouse. (**D**) Representative H&E stainings of inflammatory skin lesions. Arrows indicate dermal–epidermal clefts. Pictures were taken with 200× magnification. Scale bars represent 100 µm. Results in (**A**) were compared by two-way ANOVA and the Holm-Sidak post hoc test. Results in (**C**) were compared by unpaired *t*-test. ****, *p* < 0.0001 (n = 18 mice/group).

The antibody-transfer model of bullous pemphigoid (BP)-like epidermolysis bullosa acquisita (EBA) was conducted as previously described [27,28]. Briefly, rabbits were immunized against three epitopes of type VII collagen, and IgG directed to the epitope C (“anti-COL7c IgG”) was isolated from rabbit serum. To induce EBA, 50 μg of affinity-purified anti-COL7 IgG were injected subcutaneously on days 0, 2, and 4 of the experiment. The percentage of the total body surface affected by skin lesions was determined on the days indicated in Figure 1A. Tamibarotene was purchased from Sigma-Aldrich (Merck KGaA, Darmstadt, Germany). 3 mg/kg body weight of tamibarotene, suspended in 0.5% carboxymethyl cellulose, were administered daily by gavage in a volume of 100 μL, as previously described [4,6], starting on day 4. The control group received the vehicle only. Mice were randomized into the control and the treatment groups prior to the start of the experiment, and the control and treated mice were kept in the same cages. On days when mice were treated and EBA was evaluated, mice were first evaluated for the state of skin inflammation prior to treatment. The examiner was blinded to the treatment of the mice.

At the end of the experiment, mice were euthanized by cervical dislocation, and tissues, skin, lymph nodes (LNs), blood, and bone marrow were harvested. The treatment protocol used in this study is also summarized in Supplementary Figure S1.

### 2.3. Flow Cytometric Analyses

The cellular composition of immune cells in inflamed skin, inguinal lymph nodes, and the bone marrow at the end of the EBA experiment on day 13 were analyzed by flow cytometry.

Inguinal lymph nodes were minced and strained through a 70 μm cell strainer (Corning, Corning, NY, USA). Skin biopsies were cut into small pieces and digested using Liberase TL 400 µg/mL (Roche, purchased from Sigma-Aldrich). Afterwards, samples were spun down and resuspended in 1 mL of 1% BSA solution. Bone marrow cells were harvested as described below. Afterwards, cell suspensions then were adjusted to 10^7^ cells/mL and Fcγ receptors were blocked by FcR Blocking Reagent (Miltenyi Biotec GmbH, Bergisch Gladbach, Germany). Then, cells were stained using the antibody panels detailed in Supplementary Table S1. All stainings were analyzed on the CytoFLEX S (V4-B2-Y4-R3, Beckman Coulter GmbH, Krefeld, Germany) using the gating strategy detailed in Supplementary Figure S2. Results were analyzed using Cytoflex Cytexpert Version 2.4 (Beckman Coulter GmbH, Germany).

The effect of tamibarotene on the survival of bone-marrow neutrophils in vitro was determined by stainings with anti-annexin V-FITC (Annexin V-FITC Detection Kit, PromoKine, Catalog Number PK-CA577-K101-100) and propidium iodide.

### 2.4. Isolation of Murine Bone-Marrow Neutrophils

Bone-marrow neutrophils were isolated from the femora and tibiae of *C57BL/6J* wild-type mice. To this end, the bone marrow was flushed out using MACS buffer. The cell suspension was strained through a 40 µm cell filter (Sarstedt, Germany) and spun down using a cooled centrifuge at 400× *g* for 5 min. Erythrocytes were lysed. Cells were filtered again, spun down, and resuspended in MACS buffer. Subsequently, neutrophils were isolated by magnetic separation using Miltenyi Biotec’s (Bergisch Gladbach, Germany) Neutrophil Isolation Kit. The number of isolated neutrophils was determined using a Neubauer counting chamber. Neutrophils were resuspended in CL medium (RPMI-1640 without phenol red and with 1% FCS, 1 g/mL glucose, and 25 mM HEPES) adjusted to the desired density of the cell suspension.

### 2.5. Stimulation of Murine Bone-Marrow Neutrophils with Immune Complexes (ICs)

Fixed immune complexes were generated as previously described [29]. Briefly, immobilized immune complexes were formed using human serum albumin (Merck KGaA, Darmstadt, Germany) and rabbit polyclonal anti-human serum albumin IgG (Merck KGaA). High-binding 96-well ELISA plates (Greiner Bio one, Frickenhausen, Germany) were incubated overnight with 20 μg/mL of human serum albumin in 50 mM of carbonate/bicarbonate buffer (pH 9.6). Afterwards, wells were washed with 0.01 M of PBS (pH 7.2) and blocked for 1 h with 10% fetal calf serum in 0.01 M PBS (pH 7.2). Wells were washed again and incubated with anti-human serum albumin IgG diluted in 1/400 in PBS or with PBS only as the control for 6 h.

Bone-marrow neutrophils were freshly isolated as described above. Neutrophils were suspended in RPMI 1640 medium (Lonza, Allendale, NJ, USA) with 10% FCS and 30 µM 2-mercaptoethanol to a density of 10^6^ cells/mL and incubated with tamibarotene dissolved in DMSO in a concentration range of 3 to 3000 nM for 12 h at 37 °C. The control condition contained only the vehicle 0.003% DMSO.

To determine the response to immune complexes, 0.2 mM luminol was added to the neutrophils after 12 h, and neutrophils were seeded into the immune complex or antigen-coated wells of the 96-well plate. The plate was incubated for 2 h at 37 °C in the Infinite M200 PRO ELISA reader (Thermo Fisher Scientific), and the chemiluminescence generated by the luminol reaction, as a surrogate for the release of reactive oxygen species (ROS), was recorded. The area under the curve (AUC) of the 2 h time course of chemiluminescence was calculated using GraphPad Prism 10 (GraphPad, San Diego, CA, USA) to gauge the release of ROS over time. Afterwards, plates were spun down, and the supernatants were collected to measure the concentration of leukotriene B_4_ (LTB_4_) by the LTB_4_ Parameter Assay Kit (R&D Systems™, Wiesbaden, Germany).

### 2.6. Chemotaxis Assay

24-well Transwell plates (Sarstedt, Germany) with a pore size of the inserts of 3 µm were incubated with CL medium for 1 h at 37 °C. Then, 1 mL of CL medium with or without 10 nM of LTB_4_ was added to the bottom of the 24-well plate to assess chemotaxis. A total of 250,000 freshly isolated bone-marrow neutrophils in 200 µL of CL medium with or without 10 nM of LTB_4_, to assess chemokinesis, were added into the insert, before the plates were incubated for 2 h at 37 °C. Afterwards, the inserts were removed, and the number of neutrophils migrated into the bottom well was determined by flow cytometry using the CytoFLEX S (V4-B2-Y4-R3, Beckman Coulter GmbH, Germany) and CountBright™ Absolute Counting Beads (Thermo Fisher Scientific, Waltham, MA, USA).

### 2.7. Statistical Analyses

GraphPad Prism 10 (GraphPad, San Diego, CA, USA) was used for all statistical analyses and to analyze dose–response relationships. Unless stated otherwise, data are presented as mean ± standard error of the mean (SEM). Data were tested for normal distribution by the Brown–Forsythe test. Normally distributed data were analyzed by two-sided *t*-test or one-way ANOVA and the Holm-Sidak post hoc test depending on whether two or more groups were compared. Not normally distributed data were compared by the Mann–Whitney test or Kruskal–Wallis test and Dunn’s multiple-comparison test. Clinical scores over time were compared by two-way ANOVA and the Holm-Sidak post hoc test. The number of mice used in this study was determined by an a priori sample size calculation assuming a difference of 30% in the extent of skin inflammation between the experimental groups. No mouse subjected to the EBA model was excluded in the statistical analysis.

*p* < 0.05 was considered statistically significant throughout this study.

## 3. Results

### 3.1. Tamibarotene Aggravates BP-like EBA Markedly

To examine the impact of tamibarotene on the course of BP-like EBA, *C57BL/6JRj* wild-type mice received 3.0 mg/kg tamibarotene or its vehicle by gavage daily starting on day 4, i.e., four days prior to the first administration of anti-COL7c IgG. Mice receiving tamibarotene developed markedly more severe skin inflammation than the vehicle control group (Figure 1A,B). The aggravation of skin inflammation in tamibarotene-treated mice became evident by day 7 of the experiment when skin inflammation affected on average already 12.5% of the total body surface area of tamibarotene-treated mice but only 7.9% in vehicle-treated mice (Figure 1A). This was maintained until the end of the experiment on day 13. Thus, at this point, skin inflammation was 1.65-fold more severe in tamibarotene-treated mice than vehicle-treated mice (18.5% vs. 11.2% of the total body surface) (Figure 1A). Comparing the affected body surface at the peak of disease for each individual mouse, the data show in both groups a plateau around day 10 and inflammation in 18.9% of tamibarotene- and 11.8% of vehicle-treated mice (Figure 1C).

At the histopathological level, both vehicle- and tamibarotene-treated mice exhibited a dense infiltration of the skin with leukocytes and the formation of subepidermal clefts without any evident differences between the two groups (Figure 1D).

**Figure 2:**
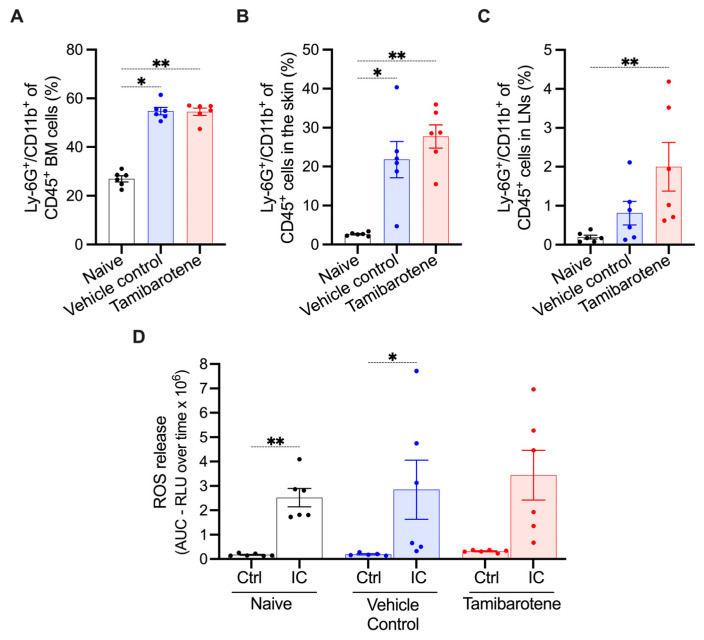
**Neutrophil numbers and their responsiveness in EBA under treatment with tamibarotene.** The percentage of neutrophils (Ly-6G^+^/CD11b^+^) among CD45^+^ cells in (**A**) the bone marrow, (**B**) (lesional) skin, and (**C**) draining lymph nodes (LNs). (**D**) ROS release of neutrophils freshly isolated from the bone marrow. All cells were harvested on day 13 of the EBA model. Under “naïve” conditions mice were not subjected to EBA and were not treated with tamibarotene or its vehicle. The interindividual variability in ROS release among the naïve group was distinctly lower than among the EBA groups, reflecting the state of chronic inflammation in these groups and its systemic effects. All results are presented as mean ± SEM. Each dot represents an individual mouse. Results were compared by the Kruskal–Wallis test and Dunn’s multiple-comparison test. *, *p* < 0.05 and **, *p* < 0.01 for the comparisons indicated in the panels (n = 6 mice/group).

As neutrophils are the predominant drivers of skin inflammation in the BP-like EBA model, we analyzed the percentage of neutrophils (Ly-6G^+^/CD11b^+^) among CD45^+^ cells in the bone marrow, in lesional skin, and in lymph nodes on day 13 in naïve mice and in vehicle- and tamibarotene-treated mice subjected to the BP-like EBA model by flow cytometric analysis. In both vehicle- and tamibarotene-treated mice, the percentage of neutrophils among CD45^+^ cells in the bone marrow had doubled compared to naïve mice, indicating that BP-like EBA had increased granulopoiesis significantly (Figure 2A). Tamibarotene did not modulate this increase (Figure 2A).

As expected, BP-like EBA substantially increased the percentage of neutrophils among CD45^+^ cells in the skin, reflecting the recruitment of neutrophils. There was also a tendency for a higher percentage of neutrophils in tamibarotene-treated mice than in vehicle-treated mice, which, however, did not reach statistical significance (Figure 2B). In lymph nodes, similarly, the percentage of neutrophils was increased in the BP-like EBA compared to naïve mice, which was increased by tamibarotene (Figure 2C).

To assess possible functional differences between neutrophils from untreated, vehicle-, and tamibarotene-treated mice, we harvested bone-marrow neutrophils on day 13, stimulated them ex vivo with fixed immune complexes (ICs), and measured the release of reactive oxygen species (ROS). The neutrophils from all three groups responded to ICs by the release of ROS (Figure 2D). There was no significant difference in ROS release between the three groups. Notably, the variability in the extent of ROS release between individual mice was much higher in the vehicle and tamibarotene groups than in the untreated group, suggesting that the inflammatory processes in the former groups might have had differential effects on the responsiveness of bone-marrow neutrophils.

### 3.2. In Vitro Tamibarotene Preserves the Responsiveness of Neutrophils to ICs

**Figure 3:**
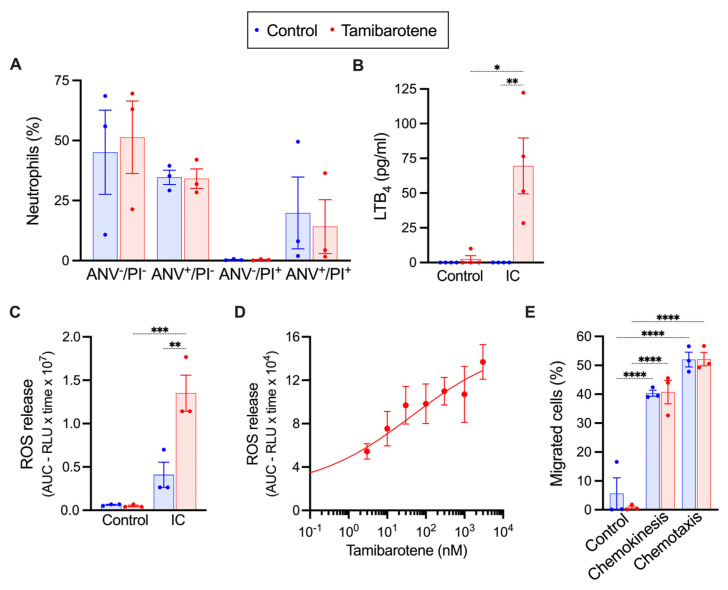
**Effect of tamibarotene treatment on neutrophils in vitro.** (**A**) Percentage of annexin V (ANV)- and propidium iodide (PI)-positive cells after 16 h of incubation with 10 nM of tamibarotene. Release of (**B**) LTB_4_ and (**C**) ROS from neutrophils upon IC stimulation after 12 h of preincubation with 10 nM of tamibarotene. (**D**) Dose-dependency of the effect of 12 h preincubation with tamibarotene on the release of ROS from neutrophils upon IC stimulation. (**E**) Chemokinesis and chemotaxis of neutrophils in response to LTB_4_. All results are presented as mean ± SEM. (**A**,**C**–**E**) n = 3 independent experiments, (**B**) n = 4 independent experiments. Dots in (**A**–**C**,**E**) represent independent experiments. Results in (**A**–**C**) were compared by the Kruskal-–Wallis test and Dunn’s multiple-comparison test and in (**E**) by one-way ANOVA and the Holm-Sidak multiple-comparison test. *, *p* < 0.05; **, *p* < 0.01; ***, *p* < 0.001, and ****, *p* < 0.0001 for the comparisons indicated in the panels.

We investigated the effect of tamibarotene on murine neutrophils freshly isolated from the bone marrow of naïve wild-type mice in vitro. First, we incubated neutrophils for 16 h with 10 nM tamibarotene and assessed the effect of tamibarotene on neutrophil survival. Tamibarotene did not alter the survival of neutrophils under these conditions. (Figure 3A). Next, we incubated neutrophils for 12 h with 10 nM tamibarotene and stimulated the cells with immune complexes (ICs) for another 2 h afterwards. After 12 h of incubation, neutrophils had become less responsive to ICs and released only minute amounts of ROS and LTB_4_ upon stimulation with ICs for 2 h (Figure 3B). Tamibarotene preserved the responsiveness of neutrophils to ICs partially. Thus, neutrophils incubated with 10 nM tamibarotene still released LTB_4_ and ROS in significant amounts (Figure 3B,C). For ROS release, we examined the dose-dependency of this effect of tamibarotene and found an almost linear dose–response relationship in a concentration range of tamibarotene of 3–3000 nM (Figure 3D). We also assessed the effect of 10 nM tamibarotene on the LTB_4_-induced chemokinesis and chemotaxis of neutrophils. Tamibarotene did not alter either (Figure 3E).

### 3.3. Tamibarotene Decreases the Recruitment of Regulatory T Cells (T_regs_) into Lesional Skin Profoundly

**Figure 4:**
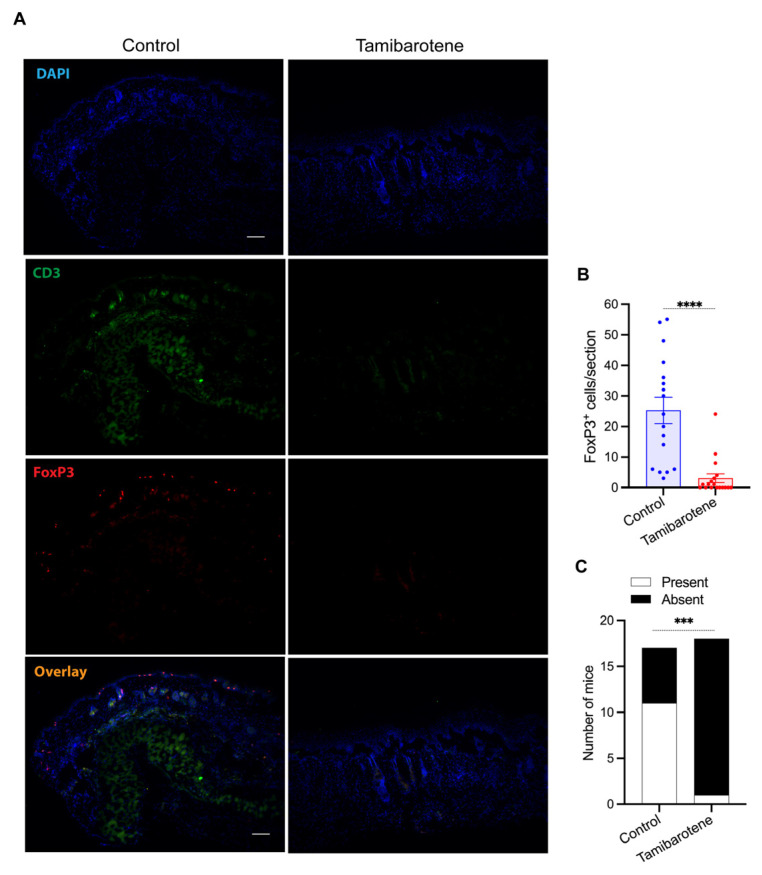
**Tamibarotene reduces the number of Tregs recruited into skin lesions in EBA.** (**A**) Representative pictures of stainings for CD3 (green) and FoxP3 (red) in lesional skin. Arrows indicate examples for FoxP3^+^ and CD3^+^/FoxP3^+^ cells adjacent to the dermal–epidermal junction. Pictures were taken with 100× magnification. Scale bars represent 100 µm. (**B**) Number of CD3^+^/FoxP3^+^ cells per section in lesional skin shown as mean ± SEM, with each dot representing an individual mouse. (**C**) Number of mice with or without chain-like accumulations of CD3^+^/FoxP3^+^ cells adjacent to the dermal–epidermal junction. Results in (**B**) were compared by the Mann–Whitney test and in (**C**) by Fisher’s exact test. ***, *p* < 0.001; ****, *p* < 0.0001 (n = 17–18 mice/group).

In our BP-like EBA model, T_regs_ are recruited to the dermis and counteract skin inflammation [20,21]. We therefore determined the number of FoxP3^+^ cells in the dermis of lesional skin by immunofluorescence staining (Figure 4A). Intriguingly, treatment with tamibarotene decreased the recruitment of T_regs_ into the dermis pronouncedly, even nullifying it in many mice completely (Figure 4B). Accordingly, while in vehicle-treated mice, FoxP3^+^ cells formed a chain-like structure close the dermal–epidermal junction, such a structure did not precipitate in tamibarotene-treated mice (Figure 4C).

## 4. Discussion

In the current study, we investigated for the first time the therapeutic potential of tamibarotene in a mouse model of an autoimmune blistering skin disease, specifically in a model of BP-like EBA. Previous studies employing models of diverse other-inflammatory diseases had demonstrated a disease-attenuating effect of tamibarotene. Anticipating a therapeutic effect of tamibarotene in this model, we surprisingly found the opposite: a marked aggravation of disease in response to tamibarotene. While vehicle-treated mice exhibited a level of skin inflammation typical for the BP-like EBA mouse model, the percentage of the total body surface in tamibarotene-treated mice reached an excessive level. Our subsequent mechanistic studies hint at two mechanisms contributing to this effect of tamibarotene in this model: a sharp decline in the recruitment of T_regs_ into the dermis and a prolonged activity of neutrophils in the skin. Both mechanisms may synergize to aggravate the neutrophilic immune response.

Tamibarotene regulates the response of multiple highly diverse cell populations in parallel. Such a promiscuity often leads to context-dependent effects of a drug. Tamibarotene exerted beneficial effects in numerous models of tissue inflammation. Most of these models depended in their pathogenesis strongly on T_H_1 or T_H_17 cells or on TGF-β-driven tissue fibrosis. The antibody-transfer model of BP-like EBA applied here differs from these models in that it primarily depends on neutrophils. This neutrophilic tissue inflammation does not essentially depend on T effector cells, but it is contained by T_regs_ at the tissue level.

Regarding skin diseases, tamibarotene was previously only tested in models of two other inflammatory skin diseases, specifically in the bleomycin-induced and in the tight skin 1 mouse model of dermal fibrosis in systemic sclerosis as well as in a bone-marrow transplantation model of graft-versus-host disease [9,10]. In the models of dermal fibrosis, tamibarotene exhibited a potent antifibrotic effect ameliorating disease by modulating pathologically relevant activities of several cell populations, especially those of dermal fibroblasts, endothelial cells, and M2 macrophages [10]. In dermal fibroblasts, it strongly inhibited the effect of TGF-β on this cell population, which is one of the central processes in the pathogenesis of dermal fibrosis. Similarly, in graft-versus-host disease, tamibarotene alleviated tissue inflammation and fibrosis exclusively in the skin by inhibiting the release of cytokines from T_H_1 and T_H_17 cells and by decreasing the expression of TGF-β [9].

Thus, in contrast to our study, these studies demonstrated a protective effect of tamibarotene. The results of these studies, however, do not contradict our results. Pemphigoid diseases differ in their pathogenesis fundamentally from that of skin inflammation in systemic sclerosis or graft-versus-host disease. While the recruitment and the actions of granulocytes in the dermis are in the center of the pathogenesis of pemphigoid diseases [30], they only play a contributory role in skin inflammation in systemic sclerosis and graft-versus-host disease. Conversely, except for certain variants of mucous-membrane pemphigoid, fibrotic processes are not involved in the pathogenesis of pemphigoid diseases.

In our in vitro studies, we investigated the effect of tamibarotene on the response of neutrophils, preincubated with tamibarotene for 12 h, to ICs. Under these conditions, tamibarotene preserved the capability of neutrophils to respond with the release of ROS and LTB_4_, which are both essential drivers for skin inflammation in the BP-like-EBA mouse model [18]. While the half-life of murine neutrophils in the blood was estimated to be 8 h, their half-life and time of activity in inflamed peripheral tissues is largely unknown and probably dependent on the specific tissue and its micromilieu. Our in vitro data suggest that tamibarotene might prolong the time neutrophils can respond to ICs. Importantly, tamibarotene did not decrease the rate of apoptosis in cultured neutrophils, pointing at other molecular mechanisms responsible for this effect. In vivo treatment with tamibarotene for 14 days, in contrast, did not increase the IC-elicited release of ROS from freshly isolated bone marrow neutrophils, supporting the notion that tamibarotene may have specifically altered the behavior of aging neutrophils in inflamed skin.

The effect of tamibarotene on neutrophils was previously investigated, and both activating and suppressive effects of tamibarotene on neutrophils were reported depending on the experimental details [1,3,31]. Among others, it was reported that tamibarotene increases the phagocytic and bactericidal activity of neutrophils from neutropenic mice [31], which aligns well with our finding that tamibarotene maintains the responsiveness of aging neutrophils to inflammatory stimuli. Another study found an inhibitory effect of tamibarotene on the response of human neutrophils to LPS and fMLP [3]. Hence, collectively, tamibarotene appears to have context-dependent effects on neutrophil responses. Additionally, a previous study reported a modest promoting effect of tamibarotene on granulopoiesis in mice under the harsh conditions of cyclophosphamide-induced neutropenia [1]. Here, we found that granulopoiesis was already strongly elevated in response to EBA, which was not further increased by tamibarotene and therefore cannot explain its proinflammatory effect in this model.

The most intriguing difference observed between tamibarotene- and vehicle-treated mice in the EBA model at the histopathological level was the almost complete abrogation of T_reg_ recruitment into inflamed skin. T_regs_ potently counteract skin inflammation locally in the dermis in our EBA [20,21]. Hence, their absence in tamibarotene-treated mice should contribute significantly to aggravating skin inflammation. Some RARs, including tamibarotene, were demonstrated to promote the differentiation of T_regs_ from naïve T cells. They were consequently suggested to play a critical physiological role, particularly in balancing T_H_17 and T_regs_ differentiation [4,32,33,34]. Importantly, they also modulate the cell-surface receptor repertoire of T_regs_. More specifically, tamibarotene and other RARs upregulate the gut-homing receptors integrin alpha4 and beta7 and the chemokine receptor CCR9 on T_regs_, while downregulating the expression of skin-homing receptors, including CCR4 [33,35,36,37]. In this project, we had not the opportunity to address the relevance of this mechanism for the effect of tamibarotene in our model experimentally, but it is a possible explanation for the abrogation of T_reg_ recruitment into the dermis under tamibarotene treatment in our mouse model.

In line with this notion, the vitamin D derivative calcitriol, which induces the expression of skin-homing receptors on T_regs_ [38,39], ameliorates disease in our EBA model [40].

Intriguingly, in bullous pemphigoid patients, the most common pemphigoid disease, the peripheral blood counts of T_regs_ are reduced compared to healthy controls and the number of T_regs_ in lesional skin is significantly lower than in lesional skin of patients with atopic dermatitis and psoriasis [41]. This collectively hints at a defect in the regulation of T_regs_ in pemphigoid diseases, possibly contributing to the eruption of inflammatory skin lesions.

In conclusion, our results show that tamibarotene abrogates the recruitment of T_regs_ into the dermis in a mouse model of pemphigoid diseases, severely aggravating disease. This finding illustrates the intricate role of RARs in the regulation of T_regs_. At this point, it is still unknown whether the results on tamibarotene generated in our mouse model are also valid for the human situation. Still, they warrant caution for the use of tamibarotene in BP-like EBA and possibly other pemphigoid diseases. Pemphigoid disease patients receiving tamibarotene should therefore be closely monitored. Furthermore, the frequency of the emergence of pemphigoid diseases under tamibarotene, as a potential side effect of this drug, should be examined.

## Data Availability

The data that support the findings of this study are available from the corresponding author upon reasonable request. Some data may not be made available because of privacy or ethical restrictions.

## Supplementary Materials

The following supporting information can be downloaded at https://www.mdpi.com/article/10.3390/cells14211661/s1: Supplementary Figure S1: Timeline of the induction of EBA, its assessment, and tamibarotene treatment; Supplementary Figure S2: Gating strategy applied to determine the percentage of neutrophils (Ly-6G^+^/CD11b^+^) in the skin, lymph nodes, and bone marrow; Supplementary Table S1: Flow cytometry panels used in this study.

## Abbreviations

The following abbreviations are used in this manuscript:

BP	Bullous pemphigoid
EBA	Epidermolysis bullosa acquisita
IC	Immune complex
