# Supplementary material for: The Retinoid Tamibarotene Aggravates Skin Inflammation in a Model of Bullous Pemphigoid-like Epidermolysis Bullosa Acquisita
Source: Cells. 2025 Oct 23;14(21):1661. doi: 10.3390/cells14211661 (PMC12607443; doi:10.3390/cells14211661)
Supplement: Supplementary file 1 [file cells-14-01661-s001.zip › cells-3887151-supplementary.pdf]

Supplementary figure S1

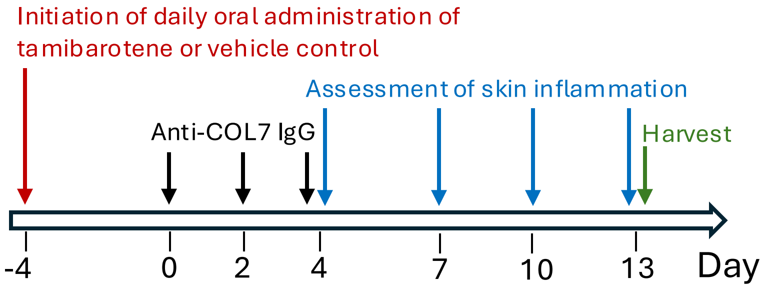

Supplementary figure S2

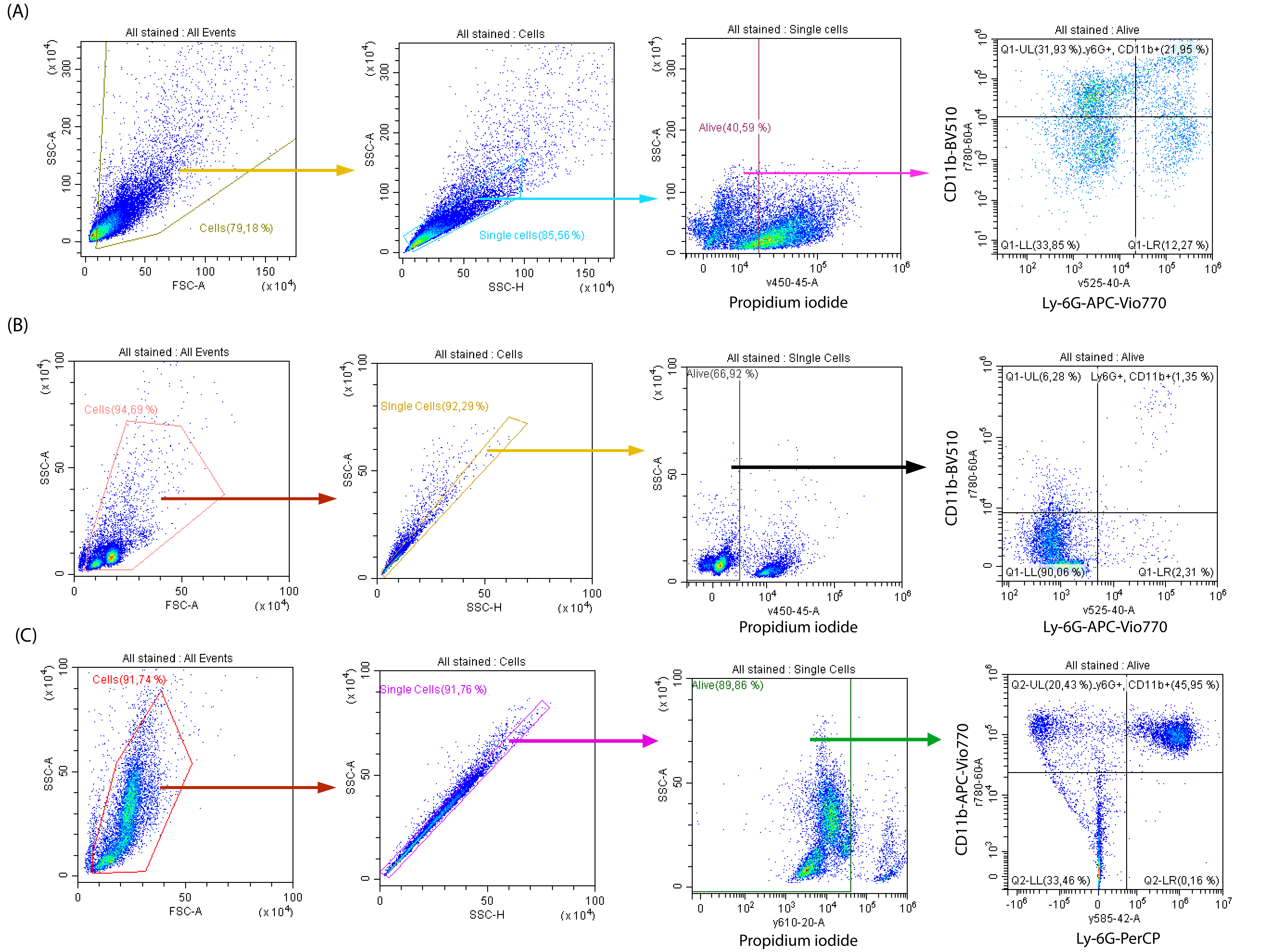

Supplementary table S1. Flow cytometry panels used in this study

Bone marrow panel		
**Marker**	**Color**	**Manufacturer/Catalog Nr.**
-	PE Zombie Red	BioLegend/423110
Ly-6G	PerCP	BioLegend/127654
CD11b	APC-Vio770	Miltenyi Biotec/130-113-232
Ly-6B	APC	Miltenyi Biotec/130-102-322
Ly-6C	PE-Vio770	Miltenyi Biotec/130-102-196
CD115	BV605	BioLegend/135517
CD117	BV421	BioLegend/105827
**Lymph node and skin panel**		
-	Vioblue	Miltenyi Biotec/130-130-404
CD11b	BV510	BioLegend/101263
Ly-6G	APC-Vio770	Miltenyi Biotec/130-118-949
CD3	FITC	Thermo Fisher Scientific/11-0031-85
CD8	BV650	BioLegend/100742
NK 1.1	APC	BioLegend/108709
CD49b	PE-Vio770	Miltenyi Biotec/130-108-176
Ly-49c	PE	Thermo Fisher Scientific/12-5991-82
